# Design of Metamaterial Absorber using Eight-Resistive-Arm Cell for Simultaneous Broadband and Wide-Incidence-Angle Absorption

**DOI:** 10.1038/s41598-018-25074-8

**Published:** 2018-04-26

**Authors:** Toan Trung Nguyen, Sungjoon Lim

**Affiliations:** School of Electrical and Electronics Engineering, Chung-Ang University, Heukseok-Dong, Dongjak-Gu, Seoul, 06974 Republic of Korea

## Abstract

In this paper, a broadband metamaterial (MM) absorber is presented for X-band applications. A novel eight-resistive-arm (ERA) cell is proposed as an MM unit cell to achieve both broadband absorption and wide incidence angles. The proposed ERA cell is designed using equivalent circuit model and full-wave analysis in order to achieve an absorption ratio higher than 90% in the range of 8.2–13.4 GHz. The experimental results indicate that the absorptivity was greater than 90% in the range of 8–13 GHz for all polarization angles under normal incidence. Under oblique incidence, the measured absorptivity was greater than 90% in the range of 8.2–12.2 GHz up to 60° and in the range of 9.2–12 GHz up to 65° in the transverse electric (TE) mode. In the transverse magnetic (TM) mode, the measured absorptivity was higher than 90% in the range of 9.5–12.4 GHz when the incidence angle was varied from 0° to 60° and remaining a 90% absorption bandwidth in the range of 10–12 GHz up to 65°. Compared to other broadband MM absorbers, the proposed MM absorber exhibited the widest incidence angles in both TE and TM modes.

## Introduction

Electromagnetic (EM) wave absorbers are applicable in various applications, including electromagnetic cloaking^1^, low-radar-cross-section materials^2^, sensing^3^, photovoltaic and thermal photovoltaic applications^4^, metal–insulator–metal^5^, and perfect absorber^6–8^. The most important applications of metamaterial (MM) absorbers are in the field of stealth technology, which is vital in the military. In general, the aim of stealth technology is to reduce signal detection or resend countermeasure signals. Therefore, researchers have attempted to reduce the scattering and reflection of radar waves from the surfaces of objects, which can be detected by radar detection systems. Radar-absorbing surfaces are used to improve the performance of stealth technology. Moreover, broadband absorption with polarization-angle-insensitive characteristics of radar-absorbing surfaces is an important feature of EM wave absorbers. Nowadays, a polarization-independent MM absorber is easily realized with a symmetric structure^9–11^. Moreover, incidence-angle-insensitivity can be achieved with a novel geometry of unit cells, such as a split-ring-cross resonator^12^, circular sector^13,14^, and surrounding via array^15^. However, the bandwidth of MM absorbers is still narrow. Many researchers have proposed alternative methods to broaden the bandwidth of MM absorbers. For instance, multi-resonators with different geometries have been placed in a one-unit cell^16,17^. Two resonators with the same geometry but different sizes have been combined^18,19^. High-absorption structures based on a composite material have been proposed^20^. An impedance layer has been added to the MM structure^21,22^ or multiple layers have been stacked^23–25^. Nevertheless, the previous broadband MM absorbers are mostly operated under normal incidence and their bandwidth becomes narrower under wider oblique incidence. For practical applications, a MM absorber must achieve high absorption under both broadband and wide incidence angle conditions.

In this paper, we introduce a novel eight-resistive-arm (ERA) cell as an MM unit cell for simultaneous broadband and wide-incidence-angle absorption. The symmetric geometry of the eight-arm resonator can facilitate the same responses at different polarization incidences. The slotted circular sector is employed owing to angle insenstivity^26^. The resistive arm is designed base on an equivalent circuit model^27,28^ to broaden the bandwidth of the MM absorber. The proposed MM absorber is realized in a single layer. Under normal and oblique incidences, the absorptivity achieved using the proposed ERA unit cell is demonstrated via full-wave simulation and measurements.

## Design and Simulation

**Figure 1:**
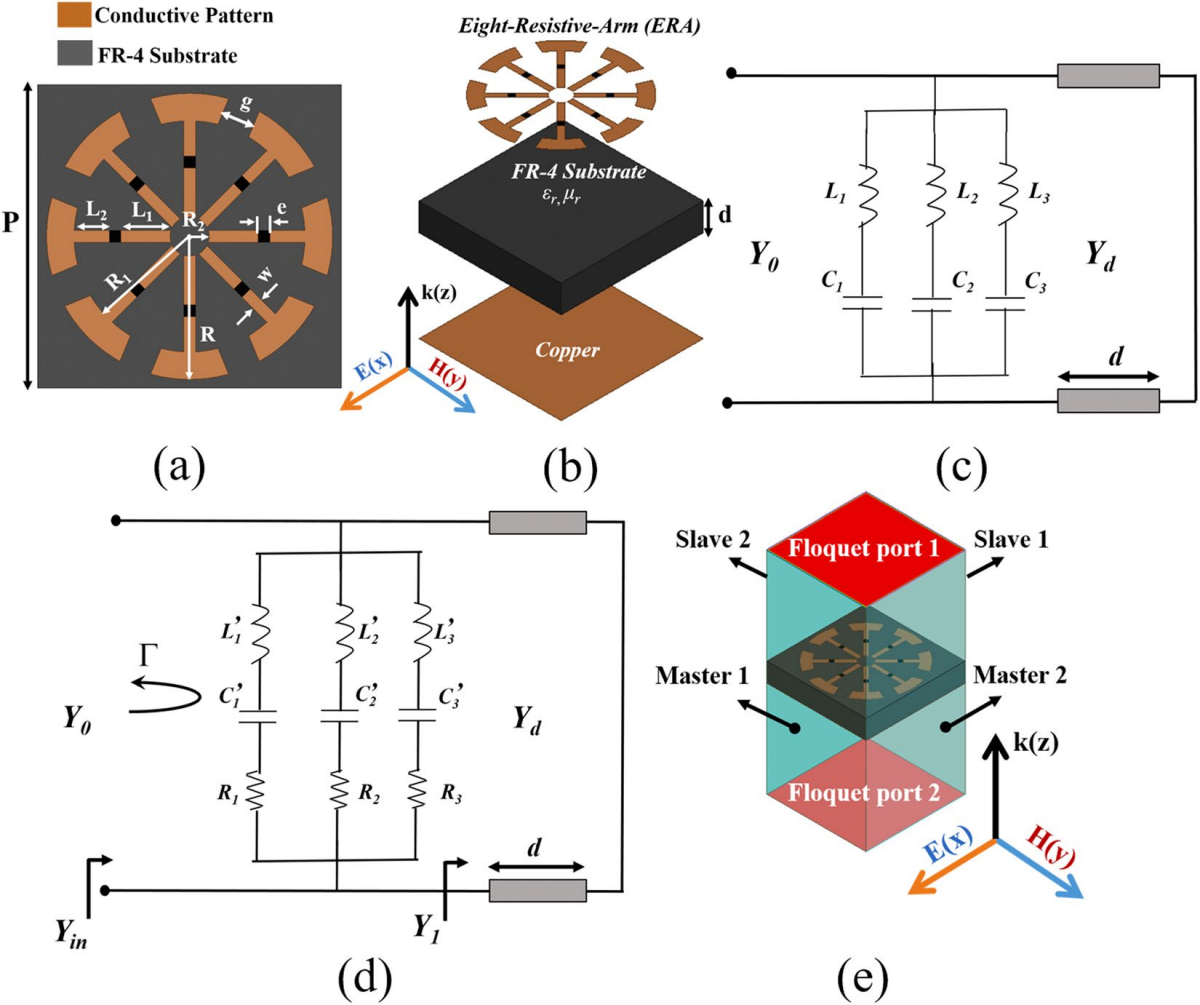
Design concept of the proposed absorber. (**a**) Top view of the proposed absorber unit cell, (**b**) 3D view of the unit cell with each layer shown separately, (**c**) transmission line model of the eight-arm unit cell without resistors, (**d**) transmission line model of the proposed ERA unit cell with resistors, and (**e**) simulation setup.

Figure 1a shows the final design of the proposed ERA as the unit cell of the MM absorber. Figure 1b illustrates the bird’s-eye view of the proposed unit cell where each layer is separately shown. The bottom plane is fully conducted as the ground to achieve zero transmission coefficient. We used ANSYS high-frequency structure simulator (HFSS) for the full-wave analysis. In order to simulate the infinite periodic array of the ERA in ANSYS HFSS, we assigned the master/slave pair as the boundary as illustrated in Fig. 1d. The Floquet ports 1 and 2 are assigned as the excitation ports. Copper, which has the conductivity of 5.8 × 10^7^ S/m, is used as the material for top pattern and bottom ground plane. The FR-4 substrate, which has the dielectric constant of 3.9 and tangential loss of 0.02, is use as the material for substrate. A general dielectric substrate has the complex relative permittivity of *ε*_*r*_  = *ε*_*r*_*′-jε*_*r*_*″* and complex relative permeability of *μ*_*r*_ = *μ*_*r*_*′-jμ*_*r*_*″*.

The transmission line models of the proposed MM absorber with and without the resistors are illustrated in Fig. 1c,d, respectively. Under normal incidence, the absorptivity A(ω) can be calculated from the equivalent circuit as

1 $$A(\omega )=1-{\rm{\Gamma }}=1-{|\frac{{Y}_{0}-{Y}_{in}}{{Y}_{0}+{Y}_{in}}|}^{2},$$

where *Y*_0_ and *Y*_*in*_ = *Y*_1_ + *Y*_*ERA*_ are the characteristic admittance of air and the input admittance of the proposed absorber, respectively. From equation (1), in order to achieve perfect absorption over the entire frequency range, *Y*_*in*_ = *Y*_0_ must be satisfied. The input admittance Y_ERA_ and Y_1_ can be calculated as follows:

2 $${Y}_{ERA}=\frac{1}{{R}_{1}+j\omega {L^{\prime} }_{1}-\frac{j}{\omega {C^{\prime} }_{1}}}+\frac{1}{{R}_{2}+j\omega {L^{\prime} }_{2}-\frac{j}{\omega {C^{\prime} }_{2}}}+\frac{1}{{R}_{3}+j\omega {L^{\prime} }_{3}-\frac{j}{\omega {C^{\prime} }_{3}}}$$

*Y*_*ERA*_ can be expressed as

3 $${Y}_{ERA}={G}_{ERA}+j{B}_{ERA},$$

where *G*_*ERA*_ and *B*_*ERA*_ are the conductance and susceptance, respectively.

4 $${Y}_{d}=\sqrt{\frac{{\varepsilon }_{0}{\varepsilon }_{r}}{{\mu }_{0}{\mu }_{r}}}={Y}_{0}\sqrt{\frac{{\varepsilon }_{r}}{{\mu }_{r}}},$$

5 $${Y}_{1}={Y}_{d}\,\coth [j\frac{\omega }{{c}_{0}}d\sqrt{{\varepsilon }_{r}{\mu }_{r}}]={Y}_{0}\sqrt{\frac{{\varepsilon }_{r}}{{\mu }_{r}}}\,\coth [j\frac{\omega }{{c}_{0}}d\sqrt{{\varepsilon }_{r}{\mu }_{r}}],$$

where *Y*_*d*_ and *ω* are the characteristic admittance and angular frequency of the dielectric substrate, respectively; *ε*_0_ and *μ*_0_ are the permittivity and permeability of free space, respectively. In order to obtain *Y*_*ERA*_, we can calculate *G*_*ERA*_ and *B*_*ERA*_ as follows:

6 $${G}_{ERA}={Y}_{in}-\mathrm{Re}({Y}_{1})={Y}_{0}-\mathrm{Re}({Y}_{1}),$$

7 $${B}_{ERA}=-\,\text{Im}({Y}_{1}).$$

Under oblique incidence, the reflection coefficients for perpendicular (Γ_⊥_) and parallel (Γ_∥_) polarizations are given by

8 $${{\rm{\Gamma }}}_{\perp }(\omega )=\frac{{Y}_{0}\,\cos \,{\theta }_{i}-{Y}_{in}\,\cos \,{\theta }_{t}}{{Y}_{0}\,\cos \,{\theta }_{i}+{Y}_{in}\,\cos \,{\theta }_{t}},$$

9 $${{\rm{\Gamma }}}_{||}(\omega )=\frac{{Y}_{0}\,\cos \,{\theta }_{t}-{Y}_{in}\,\cos \,{\theta }_{i}}{{Y}_{0}\,\cos \,{\theta }_{t}+{Y}_{in}\,\cos \,{\theta }_{i}},$$

where *θ*_*i*_ and *θ*_*t*_ are the incidence and transmission angles, respectively. Although we can design the ERA with zero reflection coefficient under normal incidence, the reflection coefficient is not zero under oblique incidence. Nevertheless, the proposed ERA has low reflection coefficients for wider incidence angles. The reflection coefficients of the ERA do not change at different polarisation angles owing to its symmetry. The resistors are loaded on the arms to broaden the absorption bandwidth.

**Figure 2:**
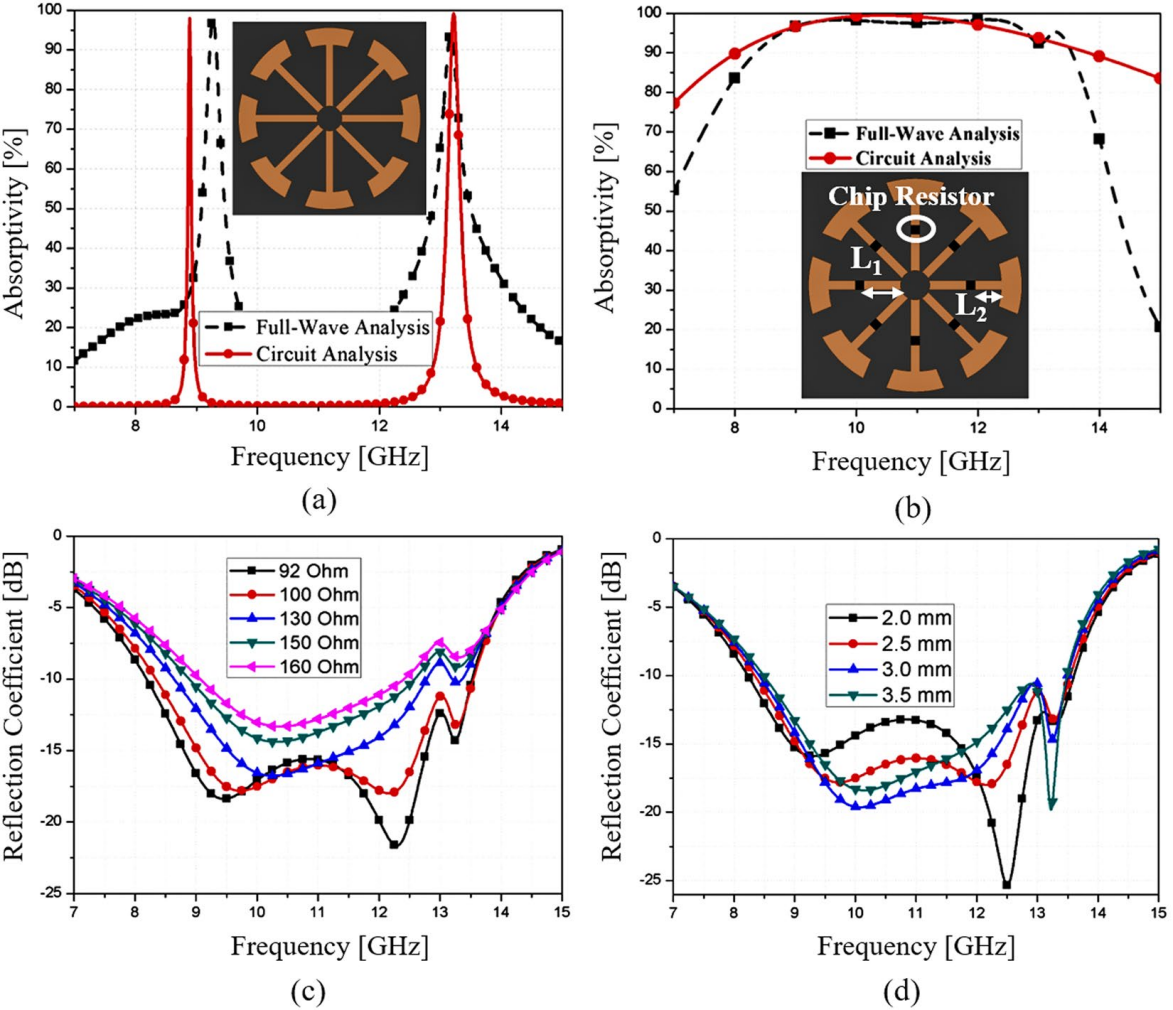
Design concept of the ERA unit cell: (**a**) simulated absorptivity of the eight-arm unit cell without resistors, (**b**) simulated absorptivity of the proposed ERA unit cell with resistors, (**c**) reflection coefficients for different resistances from 92 Ω to 160 Ω, (**d**) reflection coefficients for different locations of resistors where L1 is varied from 2.0 mm to 3.5 mm.

Figure 2 shows the design evolution of the proposed MM absorber. The proposed unit cell is based on the unit cell with the eight-arm resonator shown in Fig. 2a. The simulated absorptivity is plotted from circuit and full-wave analysis. The circuit parameters of Fig. 1c are extracted as L_1_ = 0.49 nH, C_1_ = 0.21 pF, L_2_ = 1.08 nH, C_2_ = 0.35 pF, L_3_ = 0.72 nH, and C_3_ = 0.35 pF. The absorptivity from circuit parameters is compared with the absorptivity from full-wave analysis. ANSYS high frequency structure simulator (HFSS) was used for the full-wave analysis. From circuit simulation, it exhibits two narrow resonant frequencies of 8.89 GHz and 13.2 GHz. From full-wave simulation, it exhibits two narrow resonant frequencies of 9.2 GHz and 13.2 GHz. The low resonant frequencies from the circuit and EM simulation are slightly different because the circuit parameters are extracted at 13.2 GHz. Nevertheless, the circuit analysis is useful for initial design of the unit cell. Subsequently, we loaded a resistor on each arm as shown in Fig. 2b. The circuit parameters of Fig. 1d are extracted as R_1_ = R_2_ = R_3_ = 280 Ω and L_1_′ = 0.8 nH, C_1_′ = 0.1 pF, L_2_′ = L_3_′ = 0.1 nH, C_2_′ = C_3_′ = 0.005 pF. In addition, *Y*_*d*_ is 5.24 × 10^−3^ Ω with the length of the shorted transmission line, d = 3 mm. In Fig. 2b, the absorptivity from circuit analysis and full-wave analysis are compared each other. From circuit simulation, the absorptivity is greater than 90% in the range of 8–13.5 GHz. It is observed from full-wave simulation, the absorptivity is greater than 90% in the range of 8.2–13.4 GHz.

The magnitude of reflection and bandwidth are controlled using the resistance. Figure 2c shows the simulated reflection coefficients at different resistance values from 92 Ω to 160 Ω. The optimum resistance is 100 Ω for the widest 10-dB reflection bandwidth. Furthermore, the reflection coefficients are plotted at different locations (L_1_) of the resistors when L_1_ is varied from 2.0 mm to 3.5 mm (L_2_ is varied from 2.5 mm to 0.5 mm because R_1_ is fixed to 6.1 mm). The optimum location of the resistor is 2.5 mm for the wider 10-dB reflection bandwidth.

**Figure 3:**
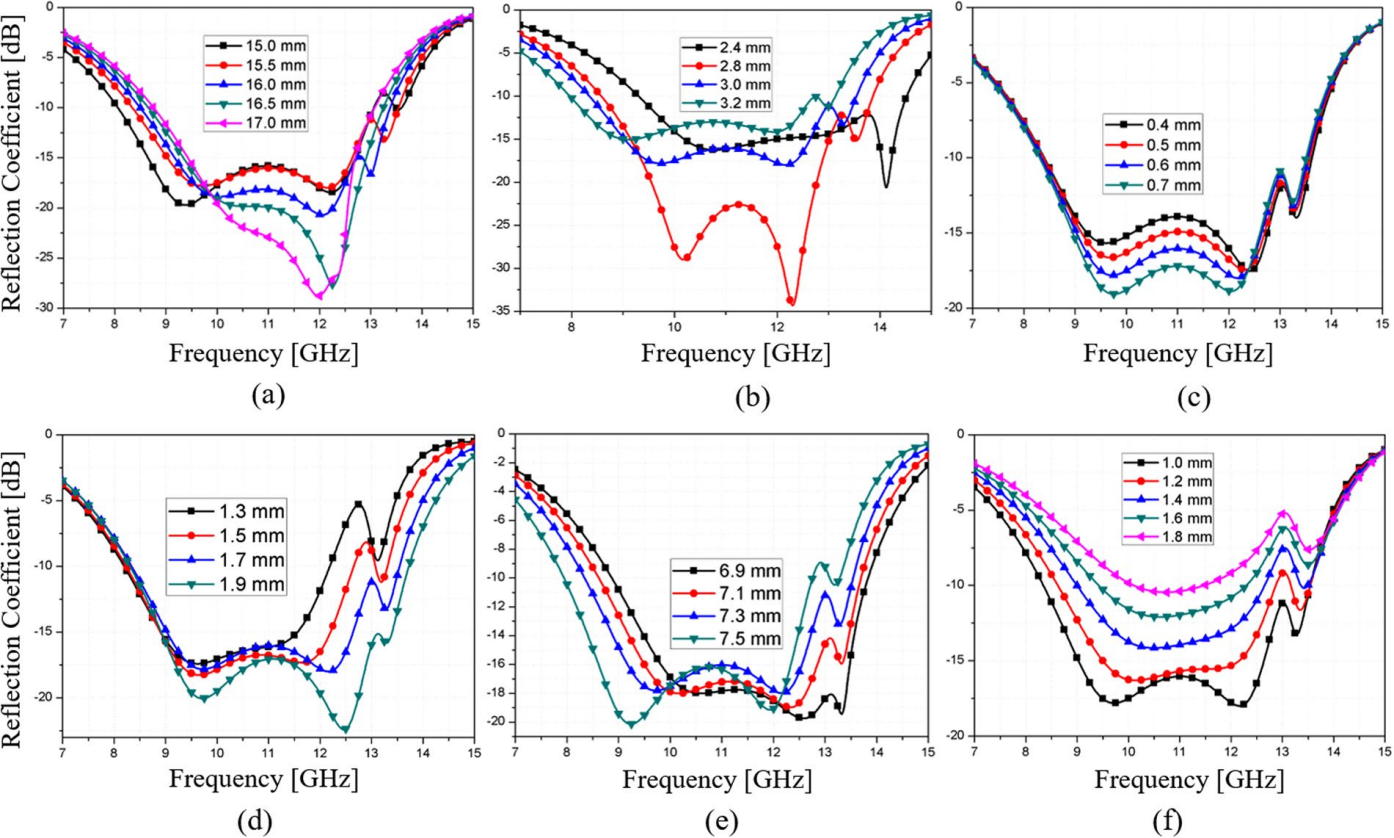
Simulated reflection coefficients for variations in geometrical parameters: (**a**) P varying from 15.0 mm to 17.0 mm, (**b**) d varying from 2.4 mm to 3.2 mm, (**c**) e varying from 0.4 mm to 0.7 mm, (**d**) g varying from 1.3 mm to 1.9 mm, (**e**) R varying from 6.9 mm to 7.5 mm, and (**f**) R_2_ varying from 1.0 mm to 1.8 mm.

Figure 3 shows the simulated reflection coefficients for variations in the geometrical parameters of the ERA. Figure 3a shows the reflection coefficients by varying the unit cell size (P) from 15.0 mm to 17.0 mm. As P is increased, the reflection coefficient in the high band becomes lower and the lowest frequency becomes higher, and thus, the bandwidth is reduced. Figure 3b shows the reflection coefficients by varying the substrate thickness (d) from 2.4 mm to 3.2 mm. Although the reflection coefficient is the lowest at d = 2.8 mm, the 10-dB bandwidth is the widest at 3 mm. Figure 3c shows the reflection coefficients by varying the gap distance for the resistor (e) from 0.4 mm to 0.7 mm. As e is increased, the reflection coefficient becomes lower. However, the gap distance must be less than 1 mm because the length of the chip resistor is 1 mm. Figure 3d shows the reflection coefficients by varying the gap distance between the outer rings (g) from 1.3 mm to 1.9 mm. As g is increased, the highest frequency is increased, and thus, the bandwidth is increased. Figure 3e shows the reflection coefficients by varying the radius of the ERA (R) from 6.9 mm to 7.5 mm. As R is increased, the absorption frequency shifts to a lower frequency. Figure 3f shows the reflection coefficients by varying R_2_ from 1.0 mm to 1.8 mm. As R_2_ is increased, the reflection coefficient becomes higher. The final dimensions of the ERA unit cell are P = 15.5 mm, R = 7.3 mm, R_1_ = 6.1 mm, R_2_ = 1 mm, L_1_ = 2.5 mm, L_2_ = 2 mm, w = 0.6 mm, e = 0.6 mm, g = 1.7 mm, and d = 3 mm.

**Figure 4:**
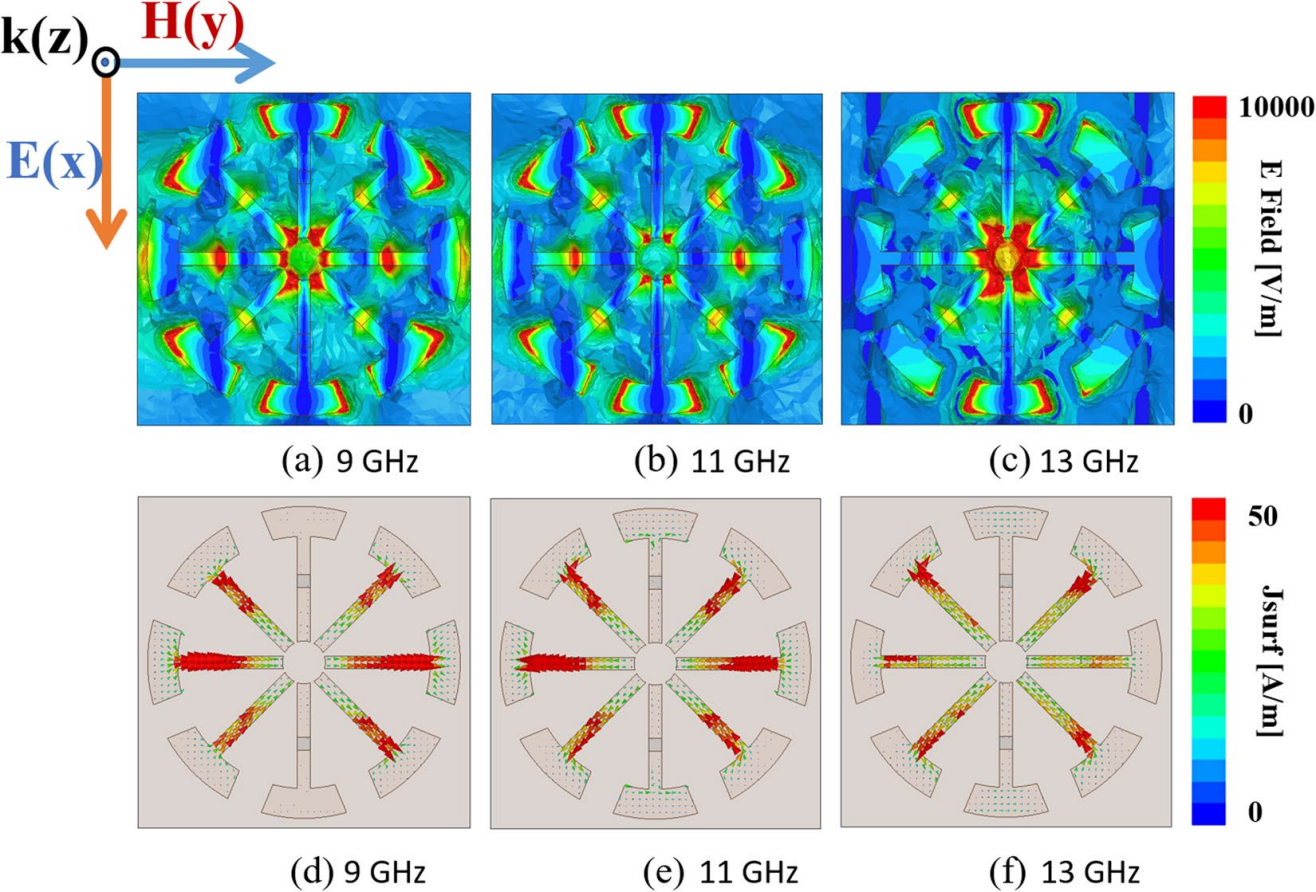
Simulated magnitude of electric field at (**a**) 9 GHz, (**b**) 11 GHz, and (**c**) 13 GHz. Simulated vector surface current at (**d**) 9 GHz, (**e**) 11 GHz, and (**f**) 13 GHz.

The electric and magnetic resonance of the proposed MM absorber can be observed from the magnitudes of the electric field and vectors of the electric current density as shown in Fig. 4. Under normal and *x*-polarized incidence, the electric field and electric current density are plotted on the XY plane of the ERA unit cells at three frequencies—9 GHz, 11 GHz, and 13 GHz. At low frequencies, the electric resonance is generated from the outer rings and resistors, whereas the electric resonance is generated from the inner ring and the vertical axis (*x*-axis in Fig. 4(c) at high frequencies. The strong electric fields on the resistors boost the bandwidth enhancement. The electric currents on the top and bottom layers are anti-parallel to each other. Therefore, the magnetic responses are generated from the electric currents on the top and bottom layers. The EM energy transmitted into the substrate is dissipated as thermal losses owing to the dielectric loss of the FR-4 substrate and the resistive losses of the resistors.

**Figure 5:**
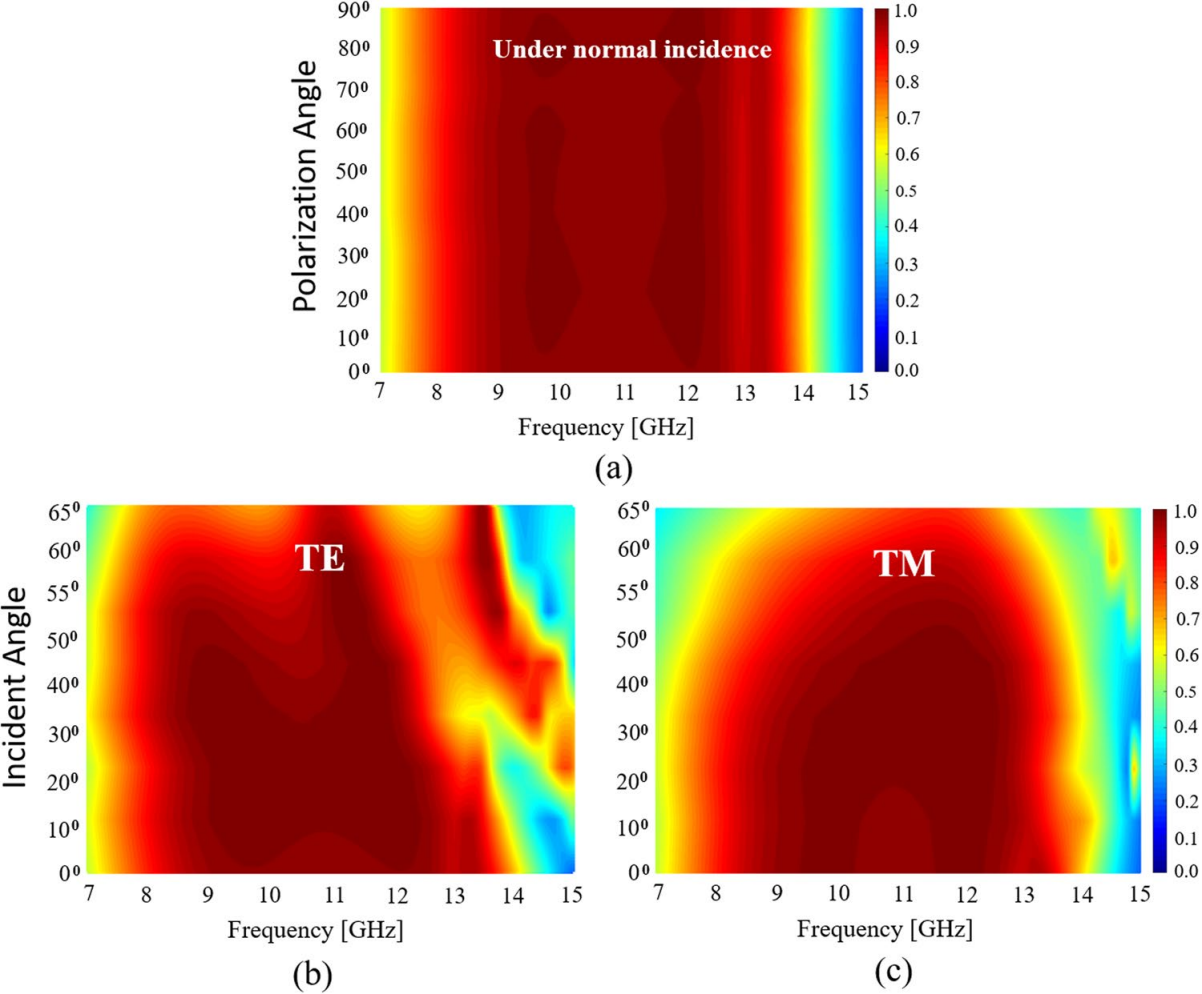
Simulated absorption spectra of the ERA according to the incidence angle of EM wave for (**a**) polarization angle (ϕ) under normal incidence, and (**b**) TE and (**c**) TM polarizations under oblique incidence (θ).

The symmetric geometry of ERA results in polarization-insensitive absorptivity. In order to demonstrate this, the absorptivity was simulated at different polarization angles, ϕ, from 0° to 90°, under normal incidence. In Fig. 5a, the absorptivity remains nearly unchanged and greater than 90% in the range of 8–13.6 GHz for different polarization angles, which confirmed the polarization-insensitivity of this absorber at normal incidence angles.

Furthermore, the absorptivity under oblique incidence was simulated by varying the incidence angle, θ, from 0° to 70° in the transverse electric (TE) and transverse magnetic (TM) modes. Figure 5b shows the gradual decrease in the absorptivity in the TE mode in the mid-band as θ increases. Nevertheless, 90% absorptivity was achieved in the range of 8.5–11.7 GHz up to θ = 60°. Figure 5c shows the significant reduction in the absorptivity in the TM mode in a high band as θ increases. This is because the direction of the magnetic field is parallel to the resistive surface layer; therefore, the electric field cannot be parallel to the layer. As observed in the TM mode, 90% absorptivity was achieved in the range of 10.5–12.3 GHz up to θ = 60°.

## Fabrication and Measurement Results

**Figure 6:**
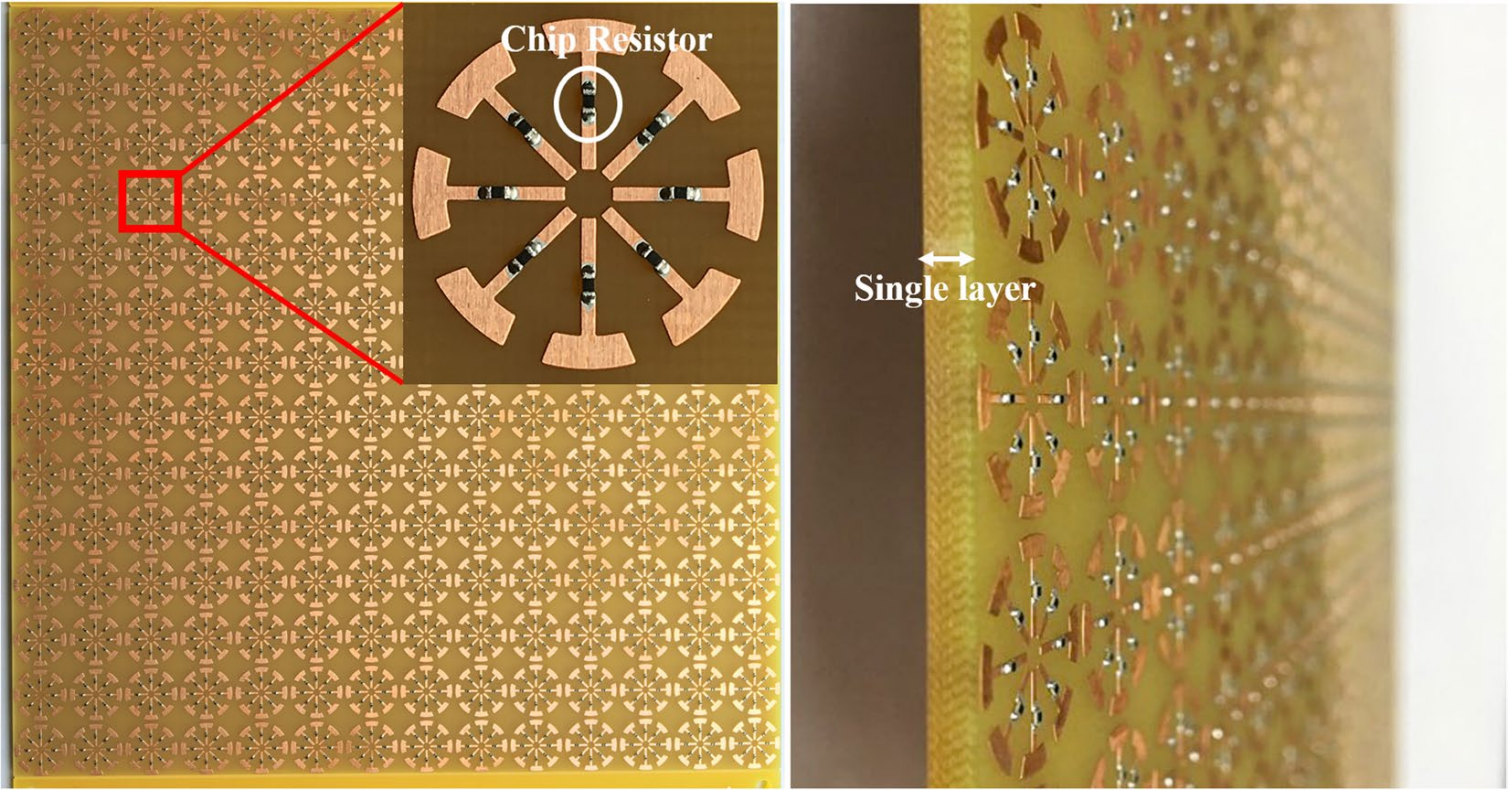
Pictures of the fabricated MM absorber prototype.

The MM absorber is fabricated using 14 × 14 ERA cells to achieve simultaneous broadband and wide-incidence-angle absorption. The prototype shown in Fig. 6 is fabricated on the FR-4 substrate with an overall size of 217 × 217 × 3 mm^3^. The conductive patterns (the top and bottom planes) are realized using copper metal. The fabricated structure consisting of 14 × 14 unit cells and 1568 100-Ω chip resistors with 1% tolerance was soldered using surface-mounting technology. We used a vector network analyser (VNA, Anritsu MS2038C) and standard-gain horn antenna (Pasternack PE9856/SF-15 WR-90) to measure the S-parameters in free space. In order to remove unwanted reflected and scattered EM waves, the absorber prototype was surrounded with wedge-tapered absorbing materials. We applied the time-gating function of the VNA to receive only the EM wave reflected from the absorber prototype. Furthermore, the measurement setup was calibrated with a copper plate of the same size as the MM absorber^14^.

**Figure 7:**
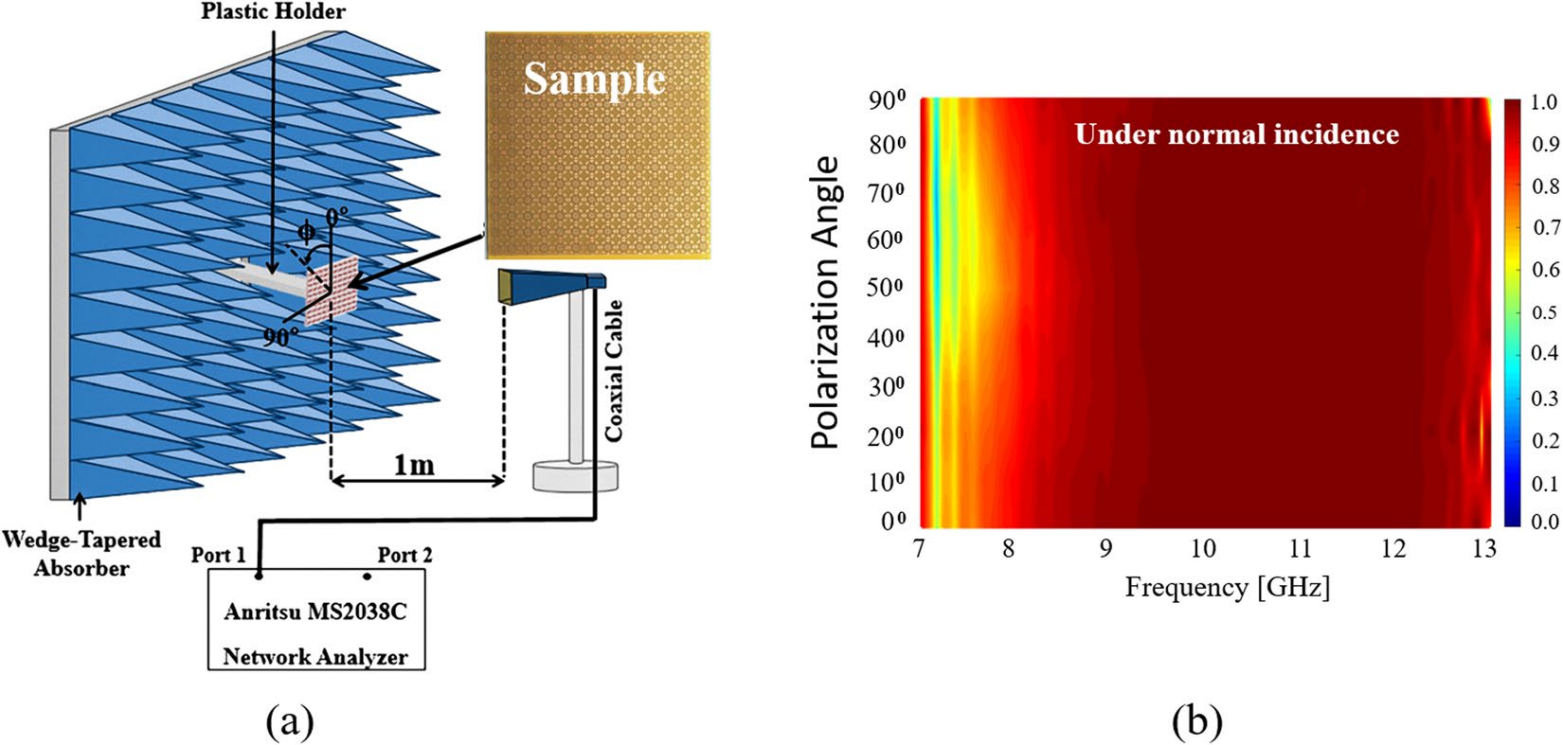
(**a**) Free-space measurement setup for normal incidence and (**b**) measured absorptivity of the ERA for polarization angle (ϕ) under normal incidence.

In order to observe the polarization sensitivity of the ERA under normal incidence, in Fig. 7a, the horn antenna is rotated with the polarization angle *ϕ* ranging from 0–90°. Figure 7b shows the measured absorptivity at the specular angle of the fabricated prototype for different polarization angles. It was observed that, for all the polarization angles under normal incidence, the absorptivity was greater than 90% in the frequency range of 8–13 GHz. It was successfully demonstrated that, under all the polarization angles, the ERA absorber showed the same absorptivity over this frequency range.

**Figure 8:**
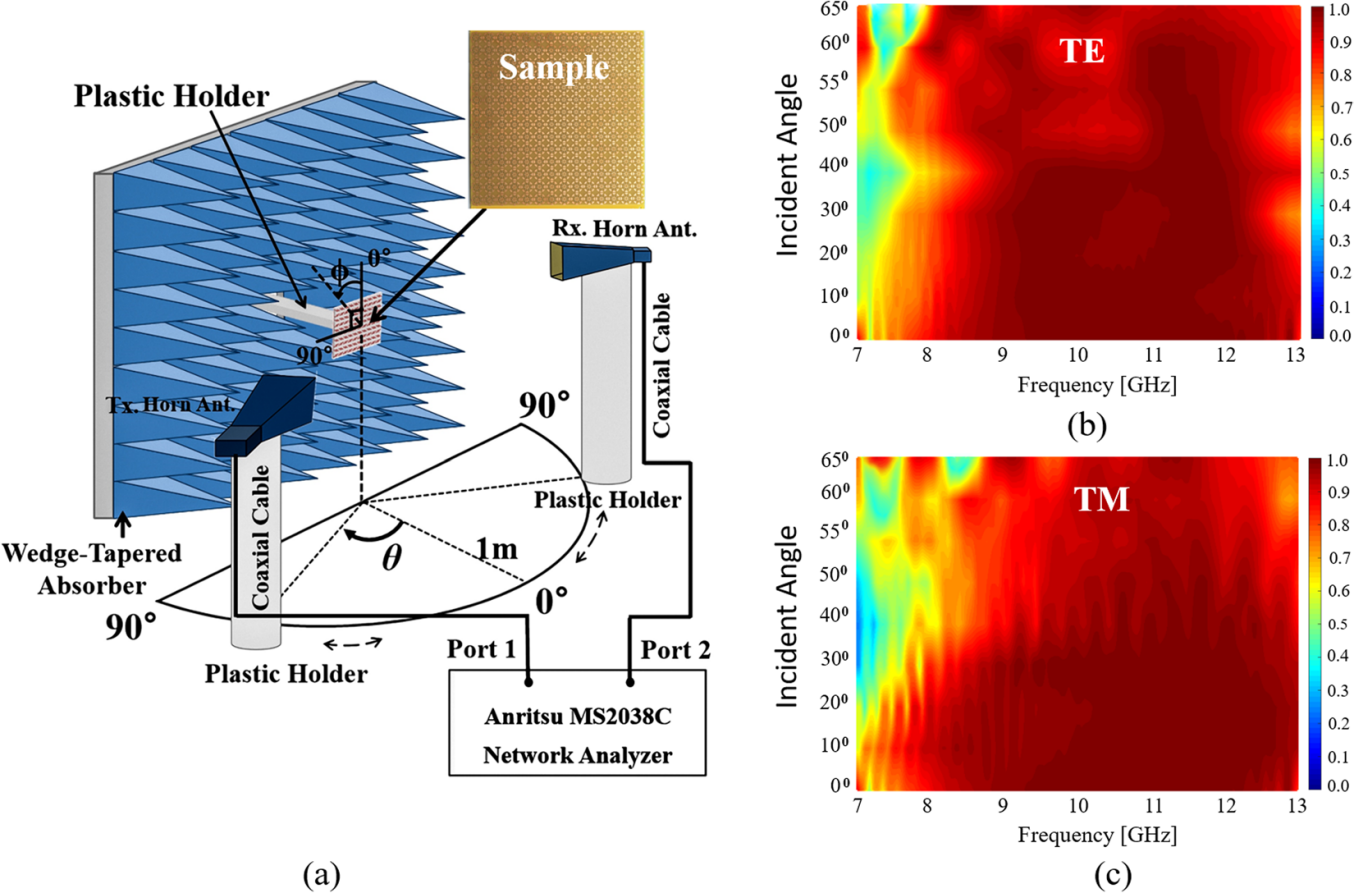
(**a**) Free-space measurement setup for oblique incidence. Measured absorptivity at the specular angle of the ERA for (**b**) TE and (**c**) TM polarizations under oblique incidence (θ).

Figure 8a shows the measurement setup for oblique incidence. The location of the transmitting horn antenna and the angle of incidence were changed by rotating the transmitting horn antenna from 0° to 70°. At each incidence angle, the receiving horn antenna was placed at the specular angle based on Snell’s reflection law and subsequently, the absorptivity was measured. Figure 8(b,c) show the measurement results of the specular angle at the TE and TM polarizations, respectively. In the TM mode, the measured absorptivity was higher than 90% in the range of 9.5–12.4 GHz when the incidence angle was varied from 0° to 60° and maintains a 90% absorption bandwidth in the range of 10–12 GHz up to 65°. In the TE mode, the measured absorptivity was higher than 90% in the range of 8.2–12.2 GHz up to 60° and in the range of 9.2–12 GHz up to 65°. The MM absorber with ERA unit cells successfully demonstrated high absorptivity at wide incidence angles of both TE and TM polarizations. Moreover, the ERA unit cells demonstrated broadband absorption.

In Table 1, the simulated and measured 90% absorption bandwidths are compared at different incidence angles for TE and TM polarization. At small incidence angles, the measured 90% absorption bandwidth was slightly narrower than the simulated bandwidth, whereas at wide incidence angles, the measured 90% absorption bandwidth was slightly wider than the simulated bandwidth. Nevertheless, the simulation and measurement results demonstrated good consistency with each other.

**Table 1:** Comparison between the simulated and measured 90% absorption bandwidths at different incidence angles.

90% absorption bandwidth [GHz]								
	*θ* = 0°		*θ* = 30°		*θ* = 50°		*θ* = 60°	
	TE	TM	TE	TM	TE	TM	TE	TM
Simulation	8.2–13.5	8.2–13.5	7.9–12.5	8.2–13.4	8–12	9.4–12.9	8.5–11.7	10.4–12.4
Measurement	8–13	8–13	8.3–12.8	8.4–13	8.5–12.5	9.5–12.8	8.2–12.2	9.5–12.4

The performance of the proposed MM absorber was compared with that of other broadband MM absorbers as listed in Table 2. The fractional bandwidth (*BW* [%]) in Table 2 is defined as

10 $$BW[ \% ]=\frac{{f}_{H}-{f}_{L}}{{f}_{C}}\times 100,$$

where *f*_*H*_ is the highest frequency in the 90% absorption bandwidth, *f*_*L*_ is the lowest frequency in the 90% absorption bandwidth, and *f*_*C*_ is the centre frequency in the 90% absorption bandwidth. The proposed absorber could achieve absorptivity higher than 90% at the widest incidence angles in both TE and TM modes. Its thinness and single layer are its additional advantages.

**Table 2:** Comparison between the performances of the proposed MM absorber and other broadband MM absorbers.

Ref.	Num. layers	f_c_ [GHz]	Thickness (mm)	*θ* = 0°		*θ* = 40°		*θ* = 60°		*θ* = 65°	
				*BW* [%]		*BW* [%]		*BW* [%]		*BW* [%]	
				TE	TM	TE	TM	TE	TM	TE	TM
^17^	1	4.8 × 10^4^	1.9 × 10^−3^ (0.03λ)	8.33	4.26	2.02	4.26	N/A	N/A	N/A	N/A
^18^	1	10	1.67 (0.056 λ)	2.06	2.06	2.11	N/A	1.05	N/A	N/A	N/A
^19^	1	10.45	0.8 (0.028 λ)	4.34	4.34	4.42	4.45	3.88	3.88	1.95	3.39
^20^	1	11.4	1.7 (0.065 λ)	115.7	115.7	N/A	N/A	N/A	N/A	N/A	N/A
^21^	1	13	3 (0.13 λ)	72	72	22.22	15.4	N/A	N/A	N/A	N/A
^22^	3	9	10 (0.3λ)	111.1	111.1	111.1	80	26.09	14.28	N/A	N/A
^23^	3	10.87	5.6 (0.2λ)	75.13	76.92	52.99	N/A	N/A	N/A	N/A	N/A
**This Work**	**1**	**11**	**3**	**51.16**	**51.16**	**41.86**	**39.21**	**37.6**	**36.36**	**26.41**	**18.18**

*N/A: Not applicable *BW [%]: 90% absorption bandwidth [%].

## Discussion

In this paper, we proposed a novel MM absorber using ERA for simultaneous broadband and wide-incidence-angle absorption. First, the eight-arm resonators are an ingenious arrangement to maintain a polarization with the vertically and horizontally symmetric structure. Second, there is a slotted circular sector between two arm resonators to keep angle-insensitive. Finally, we investigated and loaded chip resistors (100 Ω) on each arm of the ERA to broaden the bandwidth. Moreover, the design of the ERA unit cell can be understood by an equivalent circuit and its circuit parameters are calculated to realise broadband impedance matching based on the substrate and ERA pattern.

In order to demonstrate this, the ERA unit cells were fabricated on a 3-mm-thick FR-4 substrate consisting of 14 × 14 unit cells. Under normal incidence, absorptivity higher than 90% was achieved from 8 to 13 GHz for all polarization polarisation angles from 0° to 90°. Under oblique incidence, the measured absorptivity was higher than 90% from 8.2 to 12.2 GHz when the incidence angle was varied from 0° to 60° and from 9.2 to 12 GHz up to 65° in the TE mode. In the TM mode, the measured absorptivity was higher than 90% from 9.5 to 12.4 GHz when the incidence angle was varied from 0° to 60° and remaining a 90% absorption bandwidth from 10 GHz to 12 GHz up to 65°. Finally, the full-wave simulation and measurement demonstrated the polarization- and angular-insensitivities and broadband absorption of the proposed MM absorber with ERA unit cells.

From Table 2, it can be observed that the proposed ERA absorber achieved absorptivity values higher than 90% for angles of incidence up to 65° for both TE and TM polarizations. At wide angles of incidence up to 65°, the 90% absorption bandwidth of the proposed absorber was 26.41% in the TE mode and 18.18% in the TM mode, whereas almost all existing absorbers^17–23^ exhibit an approximate 90% absorption bandwidth of 0%. A previous work^19^ achieved 90% absorption bandwidths of 1.95% in the TE mode and 3.39% in the TM mode; however, these bandwidths are very small compared to the result of the present work. Moreover, the single layer and thinness of this design are additional advantages over other broadband MM absorbers.

## Method Summary

### Simulations

We used a finite-element-method-based ANSYS HFSS^19^ in our simulations. In Fig. 1b, the boundary is considered as the master/slave pair, and the excitation ports (port 1 and port 2) are considered as the Floquet ports. The ‘phi’ and ‘theta’ on the Floquet ports are defined as the polarization angle (φ) and angle of incidence (θ), respectively. By changing them, we can derive the simulated absorptivity for the polarization and incidence angles, respectively. The absorption can be calculated as $A(\omega )=1-{|{S}_{11}(\omega )|}^{2}-{|{S}_{21}(\omega )|}^{2}$, where S_11_(ω) and S_21_(ω) are the reflection and transmission parameters, respectively. As the back layer is a continuous metallic plane, S_21_(ω) ω)0; therefore, the absorption is expressed as $A(\omega )=1-{|{S}_{11}(\omega )|}^{2}$.

### Fabrication

Figure 6 shows the sample of the proposed ERA absorber. The ERA was fabricated on a 3-mm-thick FR-4 substrate with a size of 217 mm × 217 mm, which was sufficiently large to include the main beam of the transmitting antenna. The eight arms were precisely fabricated using the printed circuit board technique. The chip resistors (RES SMD 0 Ω JUMPER 1/16 W 0402 and 100 Ω) were soldered using surface-mounting technology.

### Measurement

The experimental setup of measurement is shown in Figs 7a and 8a. The absorptivity was calculated from the S-parameters. In order to measure the S-parameters, a VNA (Anritsu MS2038C) and a standard-gain horn antenna (Pasternack PE9856/SF-15 WR-90) were used. The distance from an antenna to the device under test (DUT) was investigated and maintained at 1 m to satisfy the far-field condition. In order to receive only the EM wave reflected from the absorber (DUT), we applied the time-gating function of the VNA. Moreover, to derive the S-parameters of the DUT, we used a calibration process^13,14^ as the first step to measure the S-parameters of a copper plate of the same size as the absorber sample. Subsequently, we set the magnitude of its reflection coefficient to 1 for calibration.
